# Strong DNA deformation required for extremely slow DNA threading intercalation by a binuclear ruthenium complex

**DOI:** 10.1093/nar/gku859

**Published:** 2014-09-22

**Authors:** Ali A. Almaqwashi, Thayaparan Paramanathan, Per Lincoln, Ioulia Rouzina, Fredrik Westerlund, Mark C. Williams

**Affiliations:** 1 Department of Physics, Northeastern University, Boston, MA 02115, USA; 2 Department of Physics, Bridgewater State University, Bridgewater, MA 02324, USA; 3 Department of Chemical and Biological Engineering, Chalmers University of Technology, Gothenburg SE-41296, Sweden; 4 Department of Biochemistry, Molecular Biology, and Biophysics, University of Minnesota, Minneapolis, MN 55455, USA

## Abstract

DNA intercalation by threading is expected to yield high affinity and slow dissociation, properties desirable for DNA-targeted therapeutics. To measure these properties, we utilize single molecule DNA stretching to quantify both the binding affinity and the force-dependent threading intercalation kinetics of the binuclear ruthenium complex Δ,Δ-[μ‐bidppz‐(phen)_4_Ru_2_]^4+^ (Δ,Δ-P). We measure the DNA elongation at a range of constant stretching forces using optical tweezers, allowing direct characterization of the intercalation kinetics as well as the amount intercalated at equilibrium. Higher forces exponentially facilitate the intercalative binding, leading to a profound decrease in the binding site size that results in one ligand intercalated at almost every DNA base stack. The zero force Δ,Δ-P intercalation *K_d_* is 44 nM, 25-fold stronger than the analogous mono-nuclear ligand (Δ-P). The force-dependent kinetics analysis reveals a mechanism that requires DNA elongation of 0.33 nm for association, relaxation to an equilibrium elongation of 0.19 nm, and an additional elongation of 0.14 nm from the equilibrium state for dissociation. In cells, a molecule with binding properties similar to Δ,Δ-P may rapidly bind DNA destabilized by enzymes during replication or transcription, but upon enzyme dissociation it is predicted to remain intercalated for several hours, thereby interfering with essential biological processes.

## INTRODUCTION

When synthesizing DNA-targeted drugs, high DNA binding affinity and slow DNA binding kinetics are considered essential aspects that enhance their therapeutic capability (1–5). Higher affinities maximize the treatment effect with minimal dose exposure, lower association rates enable selective binding molecules to scan DNA for a specific targeted sequence, and lower dissociation rates ensure disruption of DNA transcription and duplication (1,3,4,6–9). These desired properties are found in ligands that intercalate DNA by threading (10–13), an interaction that first requires non-intercalating moieties of a ligand to pass between DNA base pairs before the intercalative binding occurs (6,7,14,15). The threading step is required for the ligand to bring the intercalating moiety in close proximity to the base pairs in order to reach an equilibrium state, resulting in a very slow binding process (6,14,16,17). This is in contrast to conventional intercalation, which occurs by simple insertion between the DNA base pairs.

**Figure 1.**
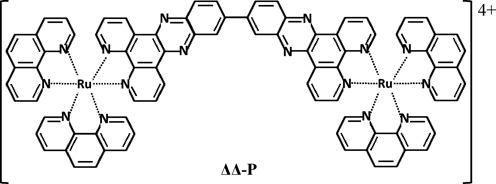
Chemical structure of the binuclear ruthenium complex Δ,Δ-P (Δ,Δ-[μ‐bidppz‐(phen)_4_Ru_2_]^4+^).

First synthesized more than a decade ago (18), the binuclear ruthenium complex Δ,Δ-[μ‐bidppz‐(phen)_4_Ru_2_]^4+^ (Δ,Δ-P) (Figure 1) exhibits extremely slow DNA intercalation kinetics, as determined by luminescence and circular dichroism (CD) bulk experiments, due to the threading intercalation binding mechanism (6,12,19). Early bulk studies overestimated the dissociation half-life of Δ,Δ-P from calf thymus DNA due to catalytic interactions by the surfactant molecules sodium dodecyl sulfate (SDS) (11,20). A revised method using competitive DNA binding reported a dissociation half-life of 38 h at 37°C, two orders of magnitude slower than the SDS measurements (11). Furthermore, Δ,Δ-P has been shown to have a preference for AT-rich DNA sequences (7,21–23). The equilibrium dissociation constant for the DNA-Δ,Δ-P complex and the molecular mechanism governing the threading intercalation have not been quantitatively determined, due to the limitations of traditional techniques for investigating ligands with extremely slow kinetics and high binding affinities.

Binding by intercalation increases the equilibrium separation between the base pairs, providing a direct measurement of DNA-Δ,Δ-P complex formation (4,10,24–27), e.g. by using single molecule DNA stretching. However, such measurements are typically performed with pulling rates much faster than the time required for slow DNA binding ligands to equilibrate (10,24). Instead, the elongation can be mechanically probed for a single DNA molecule at constant applied force while the intercalation interaction approaches equilibrium (4,10,24). Optical tweezers are able to resolve single DNA molecule intercalation elongation of a few nanometers while maintaining an applied constant force with 1 pN resolution (10,24,26,28–30). Paramanathan *et al.* demonstrated the ability of such an approach to characterize the extremely slow process of DNA-Δ,Δ-P threading intercalation at a Δ,Δ-P concentration of 2 nM (10), showing that higher forces exponentially facilitate this process. However, this study did not explore the equilibrium binding or the concentration- and force-dependent individual forward and reverse threading processes. We later developed an optical tweezers-based approach that allows complete characterization of very slow intercalation processes, and applied it to characterize the activity of the historically first anticancer antibiotic Actinomycin D (ActD) (24). Based on our findings we suggested a novel mechanism for the anticancer activity of this drug involving its ability to bind transcription-destabilized DNA exponentially stronger and faster than stable chromosomal DNA, and to remain intercalated on a very long timescale (24). The current study utilizes the same approach toward complete characterization of DNA threading intercalation by Δ,Δ-P.

## MATERIALS AND METHODS

**Figure 2.**
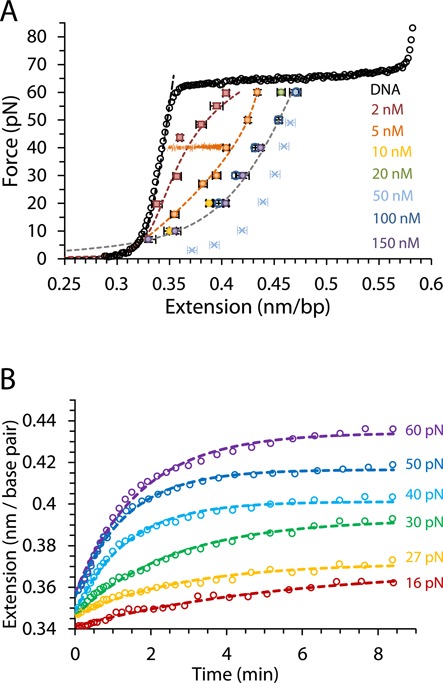
(**A**) Average measurements of at least three DNA-ΔΔ-P complex equilibrium extensions for a range of constant forces between 7 and 60 pN at ligand concentrations of 2–150 nM are shown as color coded symbols, while an illustration of a constant force measurement approaching the equilibrium extension at 40 pN and 5 nM is also shown in orange. Circles are measurements carried out in this study for concentrations 5–150 nM and squares are previously reported measurements at 2 nM (10). Cyan crosses show non-equilibrium extensions for forces 5–50 pN obtained from averaging fast release curves (obtained with a release rate of 200 nm/s) after equilibrium extension measurements with constant force (60 pN) at 50 nM concentration. Dashed lines are fits to the DNA-ligand complex WLC model in Equation (3). (**B**) The time-dependent extension as a function of time (open circles) for DNA in the presence of 5 nM Δ,Δ-P at several constant forces. The dashed lines are fits to the single exponential dependence in Equation (1). Experiments were conducted at 21°C (10 mM Tris buffer, 100 mM NaCl, pH 8).

In all experiments, dual-beam optical tweezers (lasers wavelength 830 nm) were used to trap a streptavidin-coated ∼5.6 μm polystyrene bead with a typical optical trap stiffness of 80 pN/μm, while a second identical bead was held by a micropipette with a ∼1 μm tip. A single bacteriophage λ-DNA, labeled on opposite strands with biotin, was attached between the two beads and a piezoelectric positioner controlled the micropipette displacements (±10 nm) to maintain a fixed applied force (±1 pN) on the DNA molecule (26). After the attachment between the two beads, the DNA-only stretching curve (Figure 2, black curve) is first measured at a pulling rate ∼200 nm/s. Then, the DNA is stretched rapidly (∼2 s) to reach the targeted constant force and the elongation from the DNA-only equilibrium extension due the threading intercalation by Δ,Δ-P is recorded until the DNA-Δ,Δ-P complex reaches its equilibrium extension. Typically the force feedback measurements were obtained every 200 ms, while the force feedback system can react to force changes as fast as 50 ms by mechanically repositioning the micropipette to recover the targeted force. The experiments were conducted in a flow cell chamber volume of ∼100 μl while continuously flowing solution of the desired Δ,Δ-P concentration at a flow rate of ∼2 μl/s, holding the force constant. Constant force measurements of at least three DNA molecules were acquired at each force and Δ,Δ-P concentration. All experiments were carried out at 21°C and buffer conditions of 10 mM Tris, 100 mM NaCl and pH 8. The Δ,Δ-P complex was synthesized as described elsewhere (14).

## RESULTS

### Equilibrium extension measurements at fixed force yield DNA-Δ,Δ-P titration curves and saturated complex elasticity

Equilibrium DNA extension measurements were performed for forces ranging from 7 pN to 60 pN at ligand concentrations from 5 nM to 150 nM, as shown in Figure 2A along with the previously reported measurements at 2 nM (10). Throughout the measurements, the force was maintained constant (±1 pN), which is illustrated in Figure 2A for a 40 pN measurement at 5 nM Δ,Δ-P concentration. At each constant force the extension of the DNA-Δ,Δ-P complex increased exponentially with time and converged to an equilibrium value, *L*_eq_(*F,C*), (per bp) (Figure 2B). The time evolution of the DNA-Δ,Δ-P complex extension at all forces is well fit by a single exponential with a net relaxation rate, *k*_total_(*F, C*) (4,24):

(1) \begin{equation*} L(t) = L(0) + [L_{{\rm eq}} - L(0)] \cdot (1 - e^{ - k_{{\rm total}} t} ), \end{equation*}

supporting a single molecular mechanism for threading intercalation, at least under our force-facilitated conditions. This confirms previously reported results from optical tweezers experiments for λ-DNA, and it is in agreement with bulk CD measurements for AT-DNA (10,19). However, previous luminescence measurements and CD measurements on mixed sequence DNA were fitted with multiple exponential equations, indicating multiple step behavior for Δ,Δ-P binding. It is possible that such bulk observations, which were made under very low applied forces, reflect more subtle non-threading processes (19). Such non-threading processes may include pre-intercalation binding to dsDNA, ligand rearrangements due to DNA-DNA contacts, as well as post-intercalation DNA-ligand interactions that do not contribute to DNA elongation. In contrast, the rate that is found in single DNA molecule experiments is measured exclusively from the mechanical length change due to intercalation on the timescale of tens of minutes.

For each constant force we obtained extension measurements at increasing concentration until we reached the saturated extension, *L*_sat_(*F*). The equilibrium extensions per bp of the DNA-Δ,Δ-P complex, *L*_eq_(*F,C*), are presented in Figure 2A for several force values between 10 and 60 pN. For each force the fractional equilibrium binding of ligand $\Theta (F,C)$, which ranges from zero to one, was determined by comparing *L*_eq_(*F,C*) to the saturated extension at the same force, *L*_sat_(*F*), (4,24):

(2) \begin{equation*} \Theta (F,C) = \frac{{L_{{\rm eq}} (F,C) - L_{{\rm DNA}} (F)}}{{L_{{\rm sat}} (F) - L_{{\rm DNA}} (F)}}, \end{equation*}

where *L*_DNA_(*F*) is the DNA extension in the absence of ligand binding. Afterward, we use $\Theta (F,C)$ as a function of force at each fixed concentration to obtain the equilibrium force-extension curves $L_{eq} (F,C)$ (and its reciprocal function *F*(*L*_eq_*,C*)). It follows from Equation (2) that the equilibrium force-extension $L_{{\rm eq}} (F,C)$ for each ligand concentration *C* can be calculated as a linear combination of *L*_DNA_ (*F*) and *L*_sat_ (*F*) weighted with the fraction of each component, governed by Θ(*F,C*), according to the relationship:

(3) \begin{eqnarray*} &&L_{{\rm eq}} (F,C) = \nonumber \\ &&\,\,\left[ {1 - \Theta \,(F,C)} \right] * L_{{\rm DNA}}^{} (F)\, + \,\Theta \,(F,C) * L_{{\rm sat}}^{} (F) \end{eqnarray*}

Fits to the experimental force-extension curves obtained by using Equation (3) are shown as dashed lines in Figure 2A. The saturated force-extension curve of the DNA-Δ,Δ-P complex, *L_sat_* (*F*), obtained from Equation (3) and fit to the worm-like chain (WLC) model of polymer elasticity yields an effective persistence length of ∼2 nm. This is significantly lower than the persistence length of the saturated DNA-monomer Δ-P complex of ∼14 nm (25), which intercalates DNA without threading.

Our observation that the complete *L*_eq_(*F,C*) curves calculated in this manner fit the equilibrium extensions obtained as a result of relaxation at each concentration supports the assumption that the intercalated DNA is indeed a mixture of the ligand-free and ligand-saturated DNA to a good approximation. It is important to note that, due to slow, force-dependent ligand binding, the equilibrium force-extension curve cannot be obtained directly, as this would require pulling on the timescale of many hours. To illustrate this point, a force-extension curve obtained after stretching the DNA to 60 pN at 50 nM, waiting at constant force until equilibrium is reached and then releasing at a rate of 200 nm/s shows very different elasticity due to the non-equilibrium nature of the stretching curve (cross symbols, Figure 2A). In principle, a very slow release (over many hours) of the DNA-Δ,Δ-P complex from its equilibrium extension at 60 pN would reflect the equilibrium extension fit obtained from Equation (3).

### Δ,Δ-P-DNA binding affinity and the site size are strongly affected by force

**Figure 3.**
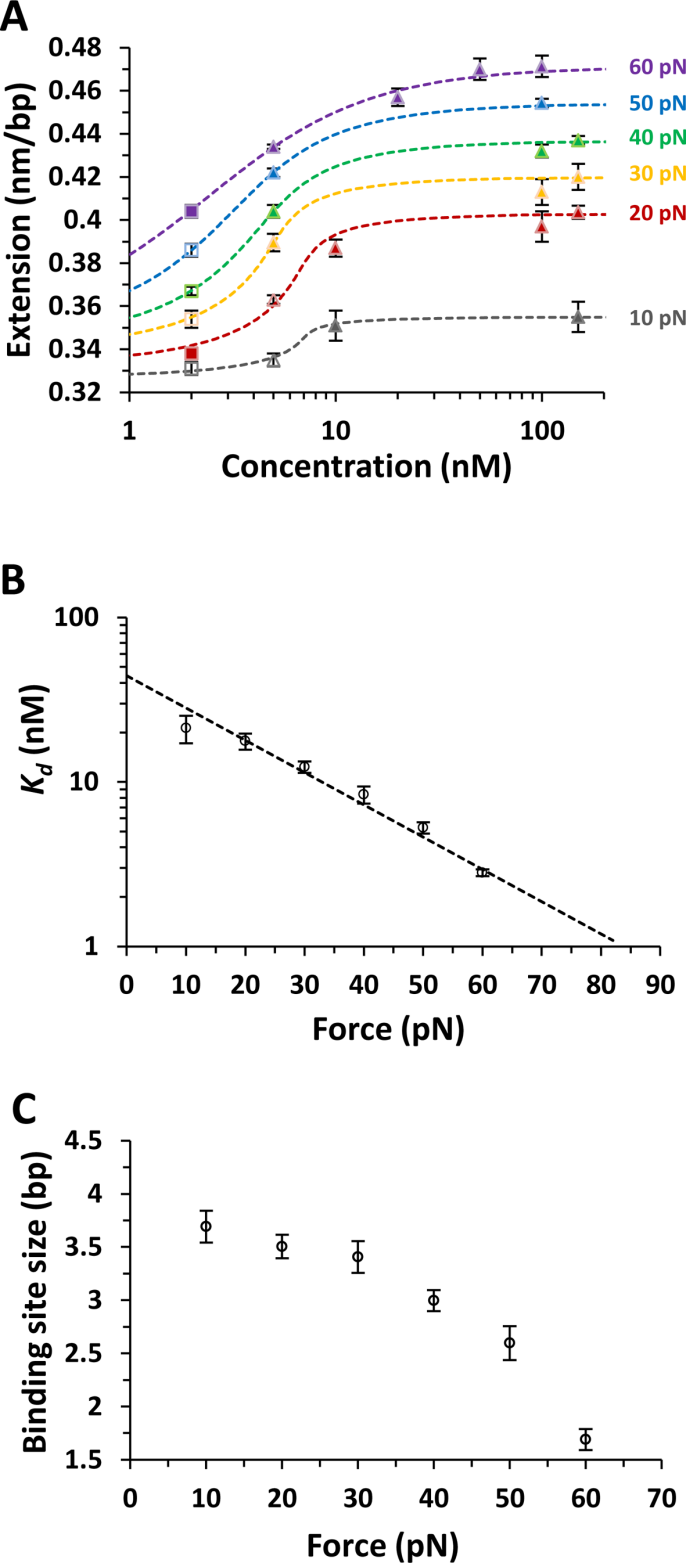
(**A**) Measured equilibrium extensions as a function of concentration fitted to the McGhee–von Hippel binding isotherm, yielding the dissociation constant (*K_d_*) and the binding site size (*n*) for a range of applied forces between 10 pN and 60 pN. The dashed lines are the fits at each constant force as color coded, closed triangles are measurements, filled squares are previously reported measurements at 2 nM (10) and unfilled marks are obtained from the DNA-ligand complex WLC fit (Equation (3)). (**B**) Measured equilibrium dissociation constant *K_d_*(*F*) (symbols) along with the fit to an exponential dependence on force (Equation 5), dashed line). The fit allows us to determine the zero force binding constant *K_d_*(0) = 44±2 nM and the change in DNA length after equilibrium binding Δ*x*_eq_ = 0.19±0.01 nm. (**C**) The binding site size obtained from the McGhee–von Hippel binding isotherm ranging from 3.7±0.2 at 10 pN to 1.7±0.1 at 60 pN.

The measured equilibrium length of the DNA-Δ,Δ-P complex *L*_eq_ (*F,C*) is presented in Figure 3A as a function of concentration. The McGhee–von Hippel binding isotherm (26,27,31–33)

(4) \begin{equation*} \Theta (K_d ,n) = \frac{C}{{K_d }}\frac{{n*(1 - \Theta )^n }}{{\left( {1 - \Theta + \frac{\Theta }{n}} \right)^{n - 1} }} \end{equation*}

is employed to fit these measurements by comparing the values obtained from Equation (2) to the fit values obtained from Equation (4), shown as dashed lines in Figure 3A. There are two fitting parameters in Equation (4), the equilibrium dissociation constant *K_d_* and the binding site size *n*, which are varied independently to fit the experimental *L*_eq_ (*F,C*) curves at each force. The binding curves could not be fit well to a single value of *n* for all curves. The fitted *K_d_*(*F*) values decrease with force from 21±4 nM at 10 pN to 2.8±0.1 nM at 60 pN (see Figure 3B). In addition, the fitted value of the binding site size of Δ,Δ-P, *n*(*F*), decreases from 3.7±0.2 at 10 pN to 1.7±0.1 at 60 pN (Figure 3C). Analyzing the fitted *K_d_* (*F*) values in terms of the expected exponential force dependence for ligand binding processes that elongate DNA (4,24,25,34)

(5) \begin{equation*} {K}_{\rm d} (F) = {K}_{\rm d} (0){\rm e}^{ - F\Delta x_{{\rm eq}} /kT} \end{equation*}

gives a zero-force equilibrium dissociation constant *K_d_*(0) of 44±2 nM. We also obtain a value of Δ*x*_eq_ = 0.19±0.01 nm, which represents the increase in DNA equilibrium extension due to one ligand binding event.

The measured force-dependent DNA-Δ,Δ-P complex elongation per bp observed at saturation, $\Delta L_{{\rm sat}} (F) = L_{{\rm sat}} (F) - L_{{\rm DNA}} (F)$, along with the fitted value of the binding site size *n*(*F*), provides an independent estimate of Δ*x*_eq_ at each force

(6) \begin{equation*} \Delta x_{{\rm eq}} = n(F)\Delta L_{{\rm sat}} (F). \end{equation*}

Substituting the fitted values of *n*(*F*) and Δ*L*_sat_(*F*) into Equation (6), we obtain the force-independent value of DNA elongation due to the single Δ,Δ-P intercalation event, Δ*x*_eq_ = 0.23±0.01 nm, averaged over all measured forces. The result is in reasonable agreement with the value of the same parameter Δ*x*_eq_ = 0.19±0.01 nm obtained by fitting *K_d_*(*F*) to Equation (5). The decreasing binding site size shown in Figure 3C implies that higher forces promote additional ligand intercalation at saturation, such that at 60 pN almost every DNA stack can be intercalated. In contrast, at 10 pN only every 3.7 stacks are intercalated at saturation.

### Kinetics analysis unveils pronounced base pair displacements required for binding

The kinetics of Δ,Δ-P binding to DNA can be followed as an increase in the DNA-ligand complex length over time, which fits well to a single exponential (Equation (1) and Figure 2B) with a net relaxation rate *k*_total_ = *k*_on_ + *k*_off_, where *k*_on_ and *k*_off_ are the bimolecular forward and the unimolecular reverse threading rates, respectively. As the DNA-Δ,Δ-P complex relaxation kinetics is determined at a fixed force, all rates pertain to that particular force. Because we also determined *K*_d_(*F*), we can use this information to obtain the individual *k*_on_(*F*) and *k*_off_(*F*) rates at each constant force (24):

(7) \begin{equation*} k_{{\rm on}} (F) = \frac{{k_{{\rm total}} (F)}}{{1 + K_d (F)/C}} \end{equation*}

(8) \begin{equation*} k_{{\rm off}} (F) = \frac{{k_{{\rm total}} (F)}}{{1 + C/K_d (F)}}. \end{equation*}

Furthermore, *k*_on_(*F*) and *k*_off_(*F*) at each ligand concentration can be individually fitted to an exponential dependence on force, which provides a measurement of the zero-force rate, *k*_on/off_(0), and the change in DNA length between the unbound and the forward transition state, *x*_on_, and the intercalated and the reverse transition state, *x*_off_, respectively,

(9) \begin{equation*} k_{{\rm on/off}} (F) = k_{{\rm on/off}} (0){\rm e}^{x_{{\rm on/off}} F/kT} \end{equation*}

**Figure 4.**
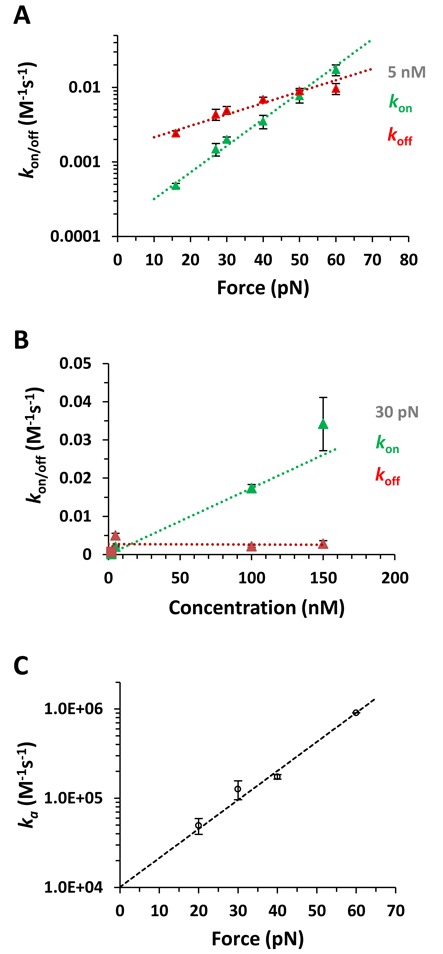
(**A**) The on rates (*k*_on_, green data points) and off rates (*k*_off_, red data points) at a ligand concentration of 5 nM fitted to a single exponential dependence on force (dotted lines) to obtain the distances associated with the on and off transitions, *x*_on_ = 0.33±0.01 nm and *x_off_* = 0.14±0.01 nm and *k*_on_(0) = 1.6±0.1×10^−4^ and *k*_off_(0) = 1.4±0.1×10^−3^. (**B**) The on rates (*k*_on_) and off rates (*k*_off_) as a function of concentration at 30 pN force. The triangles are for concentrations 5–150 nM, squares are from analyzing previously reported kinetics at 2 nM (10), and the dotted lines are linear fits. (**C**) The association rates *k_a_*(*F*) (data points) for forces from 20 pN to 60 pN fitted to Equation (9) (dashed line), yielding the zero-force association rate *k_a_*(0) = 1.01±0.001×10^4^ M^−1^ s^−1^ and the length change required for ligand association *x*_on_ = 0.31±0.01 nm.

**Figure 5.**
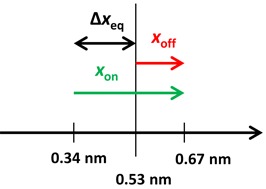
Illustration of the mechanism governing the slow kinetics of DNA threading intercalation by Δ,Δ-P, where the separation between the base pairs is nearly doubled before each association/dissociation event while the DNA-Δ,Δ-P complex relaxes at an equilibrium separation of 0.53 nm per bp.

Figure 4A presents the force-dependent kinetics analysis at 5 nM concentration. Interestingly, both *k*_on_(*F*) and *k*_off_(*F*) are strongly facilitated by force, due to the significant complex elongations required for both ligand association (*x*_on_ = 0.33±0.01 nm) and dissociation (*x*_off_ = 0.14±0.01 nm). Similar dynamic displacements are obtained from analyzing the kinetics at all other concentrations below saturated binding (data not shown). The difference between the measured complex elongations to the transition state in the forward and reverse directions provides another independent estimate of the equilibrium complex elongation upon a single Δ,Δ-P intercalation event (Figure 5), Δ*x*_eq_ = *x*_on_- *x*_off_ = 0.19±0.01 nm, which agrees with the two other measurements of this parameter discussed above.

The forward rates *k*_on_(*F*) for all measured forces show a linear dependence on ligand concentration, whereas the backward rates *k*_off_(*F*) are independent of concentration, as expected for a bimolecular process. For example, the forward rate *k*_on_ at 30 pN ranges from ∼10^−4^ s^−1^ (τ ∼2 h) at 2 nM to ∼10^−2^ s^−1^ (τ ∼2 min) at 150 nM, as illustrated in Figure 4B. The slope from the linear concentration dependence at each measured force gives the association rate *k*_a_(*F*), as shown in Figure 4C for all measured forces. By fitting the association rates to the exponential dependence on force (Equation (9)), we obtain a zero-force association rate of *k_a_*(0)∼10^4^ M^−1^ s^−1^. This bimolecular association rate is ∼5 orders of magnitude slower than an association rate limited by diffusion.

## DISCUSSION

### Equilibrium intercalation properties of Δ,Δ-P

DNA threading by Δ,Δ-P is dramatically facilitated by force, as indicated by the exponential decrease in the DNA-ligand equilibrium dissociation constant with applied stretching force (Figure 3B). This Δ,Δ-P -intercalated DNA state is stabilized by force because each intercalation event results in a DNA elongation of Δ*x*_eq_ = 0.19 nm. Also, the DNA stretching force appears to relieve significant steric constraints against Δ,Δ-P threading, leading to saturated Δ,Δ-P-DNA intercalation every 1.7 bp at 60 pN force (Figure 3C), in contrast to the observed saturated intercalation of only every ∼3.7 bp at low forces (10 pN).

In Table 1, we compare the parameters describing equilibrium Δ,Δ-P binding with the analogous parameters for the corresponding mononuclear complex, Ru(phen)_2_dppz^2+^ (Δ-P), also measured by DNA stretching with optical tweezers (25). Only the equilibrium intercalation by these two related ligands can be compared, as the rapid intercalation of the Δ-P molecules on our DNA stretching timescale ∼1 min does not allow studies of its intercalation kinetics (25). At low forces (*F* = 10 pN), the intercalative binding site size of Δ,Δ-P (3.7 bp) is somewhat larger than that of Δ-P (2.9 bp), as could be expected from the larger size of the former complex. However, increasing force has a much more dramatic effect on the binding site size of Δ,Δ-P, which decreases by 2 bp to 1.7 bp at 60 pN, relative to the binding site size of Δ-P, which decreases by only 0.4 bp (Table 1). Surprisingly, the equilibrium DNA elongation upon monomer Δ-P intercalation Δ*x*_eq_ = 0.38 nm is almost twice as large as the elongation upon Δ,Δ-P intercalation, Δ*x*_eq_ = 0.2 nm. Also, the measured zero-force equilibrium dissociation constant of the Δ,Δ-P molecule *K_d_* = 44 nM is 25-fold smaller than for Δ-P, suggesting additional interactions that drive Δ,Δ-P intercalation, despite the similar dppz stacking moiety for these two ligands. Finally, the flexibility of the equilibrium ligand-saturated DNA complex for both monomer- and dimer-ligands is much higher than for the ligand-free double-stranded B-from DNA, as described by the corresponding persistence length of these complexes of 14 nm, 2 nm and 50 nm, respectively.

**Table 1.** Comparison of the equilibrium intercalative parameters for mononuclear and binuclear ruthenium ligands

Property of intercalated complex	Δ-P Ru(phen)_2_dppz^2+^	Δ,Δ-P Δ,Δ-[μ‐bidppz‐(phen)_4_Ru_2_]^4+^
Δx_eq_ (nm)	0.38 ± 0.02	0.19 ± 0.01
*n*(*F* = 10pN)	2.9 ± 0.1	3.7 ± 0.1
*n*(*F* = 60pN)	2.5 ± 0.1	1.7 ± 0.1
*K_d_*(*F* = 0), (μM)	1.1 ± 0.1	0.044 ± 0.002
Persistence length (nm)	14.3 ± 0.1	1.6 ± 0.2
Elastic modulus, (pN)	324 ± 17	598 ± 30

These results may be interpreted in terms of the recent crystal structure of Δ,Δ-P intercalated into a double-stranded DNA 6-mer d(CGTACG) (16). According to this study, the Δ,Δ-P molecule traverses the DNA double helix and displaces an AT base pair from the DNA stack to position the two bulky Ru centers on the opposite groove sides of the double helix. These A and T bases that are displaced into the minor groove make extensive stacking contacts with the phen aromatic moieties of the Ru center located in the minor groove. The overall Δ,Δ-P-DNA binding mode was termed ‘insertion threading intercalation’, as opposed to non-displacive intercalation, which occurs for the mono-nuclear ruthenium compound Δ-P (35,36). The difference between these two types of intercalation provides a potential explanation for the difference in DNA elongation after these binding events, as observed in our DNA stretching experiments. Indeed, the classical intercalation typical of the Δ-P ligand leads to duplex elongation by ∼0.38 nm, which is similar to the length of a normal base pair stack ∼0.34 nm, as for most of the other classic intercalators (25,27,37). In contrast, the inserted aromatic dppz moiety of Δ,Δ-P takes the place of the extruded base, thereby leading to a much smaller DNA elongation. The more severe duplex deformation caused by insertion threading intercalation is also consistent with the much smaller persistence length of the saturated Δ,Δ-P-DNA complex of 2 nm observed here. While this is much smaller than the B-form DNA persistence length of 50 nm, it still involves 4–6 intercalated DNA base pairs, in agreement with the relatively straight conformation of the final equilibrium bound state, in which the DNA duplex can be described as a distorted form of B-DNA and appears to be underwound (16).

### Kinetics of DNA intercalation by Δ,Δ-P

The very slow forward and reverse threading kinetics is related to the major DNA duplex deformations required for either process. Our data suggests that the threading of Δ,Δ-P involves a rate-limiting step of DNA elongation by 0.33 nm per ligand, which might involve severe deformation including opening of one or two base pairs. Estimates of the enthalpy of this deformation obtained by studying the temperature dependence of Δ,Δ-P threading rate (11) are in the range of ΔH∼ 22–40 kcal/mol. This can be compared to the enthalpy of a single bp fluctuational opening in the middle of the duplex, which was determined based on NMR studies to be ΔH_1m_ = 10–26 kcal/mol (38). Therefore, the enthalpy of the threading deformation is likely dominated by opening of one to two base pairs. A smaller, but still significant, DNA deformation of *x*_off_ = 0.14 nm required for dissociation of Δ,Δ-P from the intercalated state, follows from our observation that the stretching force facilitates dissociation (Figure 3B). As the forward process is promoted by force more strongly than the reverse process, the overall binding is exponentially facilitated by force, in agreement with the resulting overall equilibrium displacement $\Delta x_{{\rm eq}} \approx 0.2\,{\rm nm} \approx x_{{\rm on}} - x_{{\rm off}}$. The observation that the force promotes threading intercalation by Δ,Δ-P via DNA duplex destabilization is consistent with previous solution studies, which showed that Δ,Δ-P binding to DNA is strongly facilitated by negative super-helical stress in closed circular DNA (39), by the presence of AT-rich sequences (21,39), by structural defects (40) and at higher temperatures (11,12). Moreover, the propensity of Δ,Δ-P to specifically intercalate DNA quadruplex structures with both of its dppz moieties suggested by the recent crystal structure (16) opens the possibility for Δ,Δ-P to be able to recognize specific DNA structural motifs.

The force-facilitated Δ,Δ-P-dsDNA on rates in our experiments can be well described by a single exponential dependence on time (Figure 2B). This is in contrast to the complex threading intercalation kinetics observed in bulk solution experiments with mixed sequence DNA (19). We suggest that the simple kinetics is typical of the fastest intercalation event, which dominates the process under the action of applied force. Because we see strong saturation of DNA with Δ,Δ-P during this process, this likely constitutes the major intercalative pathway. The measured bimolecular Δ,Δ-P association rate *k_a_*(0) = 10^4^ M^−1^s^−1^ is ∼5 orders of magnitude slower than the association rate limited by diffusion *k*_dif_ ∼10^9^ M^−1^s^−1^ (41). The fact that the reaction appears bimolecular implies that the first Δ,Δ-P binding step, which is known to involve the fast non-intercalative DNA-ligand binding of primarily electrostatic nature (12,20), is very unstable at ligand concentrations typical of intercalative binding *K_d_*∼10 nM, such that the ligand dissociates from this non-intercalated state much faster than it threads. The overall forward intercalative rate $k_a (0) = k_{{\rm dif}} e^{ - G_{{\rm thread}} /k_B T}$is a product of the non-intercalative bimolecular association rate *k_dif_* and the probability of intercalation during non-specific binding, $e^{ - G_{{\rm thread}} /k_B T}$, where $G_{{\rm thread}} \sim k_B T \cdot \ln (k_{{\rm dif}} /k_a (0))$ such that $G_{{\rm thread}} \sim 11.5\,k_B T = 6.8\,{\rm kcal/mol}$ is the free energy of DNA deformation involved in the rate limiting step of Δ,Δ-P threading. This free energy is consistent with the complete melting of 1–2 base pairs in the middle of the DNA duplex. While the stability of the single terminal duplex bp is only 2–4 *k_B_T*, the creation of the helix/coil boundaries associated with local duplex disruption can easily introduce an additional 8–10 *k_B_T* (42). The free energy cost of threading deformation is much smaller than its enthalpy estimated from the temperature dependence of threading, discussed above. This result is not surprising, as the large enthalpy cost of threading deformation can be partially compensated by the associated entropy gain.

*In vivo* it is likely that the very slow threading of Δ,Δ-P into stable polymeric dsDNA would prevent this ligand from intercalating most chromosomal DNA if it reaches the nucleus. However, DNA undergoing high rates of transcription and replication, such as may be found preferentially in cancer cells, may already be destabilized by RNA polymerase or other motor proteins, facilitating rapid initial binding to these locations, followed by very slow dissociation. Therefore, the slow dissociation kinetics combined with its high DNA binding affinity highlights the potential therapeutic use of ligands with binding properties similar to Δ,Δ-P (43,44).
